# The lead leads the way

**DOI:** 10.1007/s12471-021-01625-z

**Published:** 2021-08-27

**Authors:** S. Borges, J. I. Moreira

**Affiliations:** Cardiology Department, Centro Hospitalar de Trás os Montes e Alto Douro, Vila Real, Portugal

## Answer

**Fig. 1:**
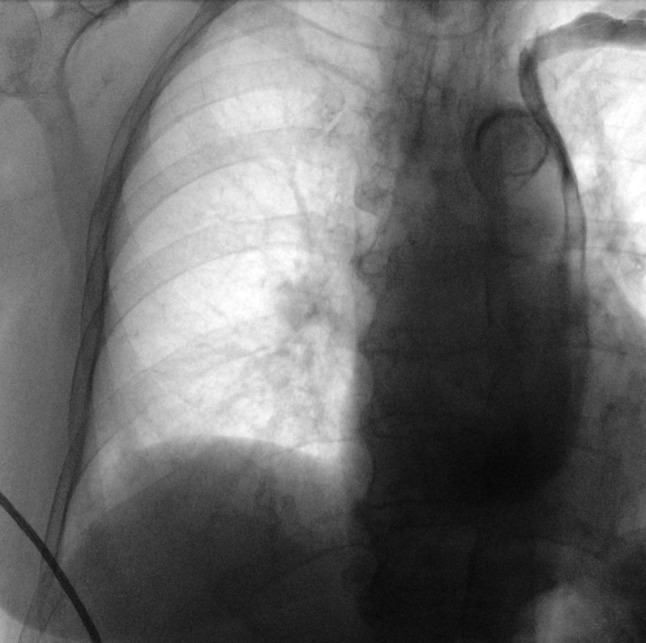
Venography of the left subclavian vein

To our surprise, the guide wire was advanced, entered the subclavian vein and descended parallel to the spine without crossing over to the right side. Thereafter, the guide wire traversed the coronary sinus (CS) and terminated in the right atrium. Venography of the left subclavian vein was performed, showing the presence of an isolated persistent left superior vena cava (PLSVC), with absence of the innominate vein (Fig. 1).

PLSVC is a rare congenital vascular anomaly, occurring in 0.3–0.5% of individuals in the general population [1]. These challenging anatomical variants, often asymptomatic and an incidental finding at the time of the procedure, can pose difficulties and complications during central venous cannulation or device implantation, such as arrhythmia, cardiac tamponade, CS dissection and thrombosis. Additionally, lead insertion may be technically challenging due to misalignment of the CS ostium in relation to the tricuspid valve [2].

However, the development of new materials and techniques in recent years has enabled the successful and safe implantation of cardiac devices [2]. Our patient remains asymptomatic and pacemaker interrogation revealed normal parameters during the follow-up period.

Even though permanent pacemaker implantation through the CS via the PLSVC is technically demanding, long-term results are good [3], as observed in our case. However, these patients need frequent surveillance during the initial period.
